# Single-Sample Melt-Based Screening for Rifampicin Susceptibility in the Emerging Mutation Hotspot at *rpoB* Codon 491

**DOI:** 10.1021/acsinfecdis.5c00150

**Published:** 2025-06-17

**Authors:** Nicole A. Malofsky, Swayashreyee B. Dhungel, Megan E. Pask, Frederick R. Haselton

**Affiliations:** Department of Biomedical Engineering, 5718 Vanderbilt University, Nashville, Tennessee 37235, United States

**Keywords:** melt analysis, mutation screening, emerging hotspot, tuberculosis, rifampicin resistance, L-DNA

## Abstract

Based on sequencing data, mutations at *rpoB* codon 491 ofare associated with rifampicin resistance, but current commercial and WHO-endorsed genotypic tests fail to detect them. As a result, resistant infections go untreated, driving transmission and multidrug resistance. A real-time PCR assay by André et al. specifically screens for I491F but omits other codon 491 mutations. To address this gap, a single-sample screening method using asymmetric PCR followed by melt analysis was developed for the three sequence-identified variants, I491F/N/M. Each sample contained a melt probe matching the susceptible sequence, which, after asymmetric PCR spanning codon 491, hybridized with the excess strand to form a duplex. The duplex’s melt temperature (*T*_m_) was then measured. To enable single-sample classification, each reaction also included double-stranded L-DNA identical to the probe and wild-type PCR product duplex. Susceptibility was determined by the within-sample *T*_m_ difference between the probe-product and L-DNA duplexes. The approach was evaluated and compared to the André assay across two calibrated PCR instruments using synthetic *rpoB* wild-type and variant sequences. As expected, the André assay distinguished wild-type from I491F samples but misclassified I491N and I491M samples based on multisample *T*_m_ comparison. In contrast, our single-sample classification strategy used within-sample *T*_m_ differences, classifying samples as rifampicin-susceptible when the within-sample *T*_m_ difference was less than 0.83 °C. With this approach, the method achieved 100% sensitivity and 100% specificity across both PCR instruments. Although demonstrated for *rpoB* codon 491, this assay design is readily adaptable to any other sequence-identified, clinically significant mutation hotspot.

## Graphical abstract

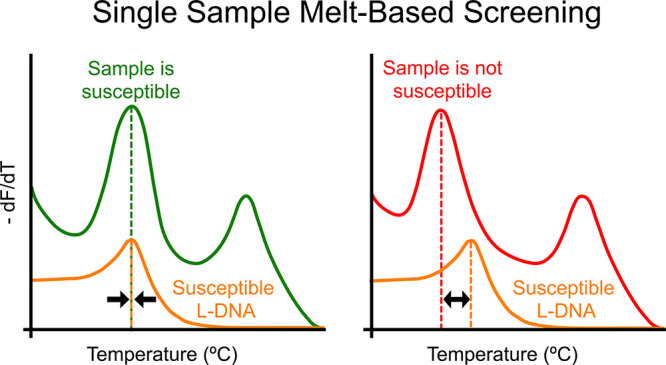

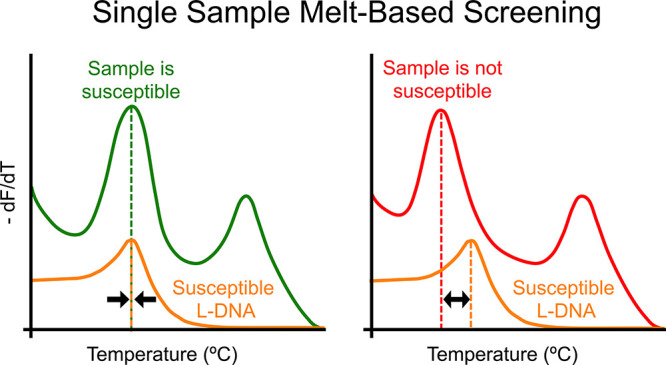

The global burden of tuberculosis (TB) is exacerbated by the emergence of drug-resistant strains with mutations that render drug treatments ineffective.^1^ In particular, mutations associated with rifampicin resistance pose a significant challenge to TB control by compromising the efficacy of rifampicin, the most potent first-line anti-TB drug.^2,3^ Each year, approximately half a million people are infected with rifampicin-resistant TB, creating a significant barrier to disease management and control.^2^ Rapid and accurate drug susceptibility testing is critical to ensuring that patients receive the most effective treatment regimens and reduce the spread of drug-resistant TB strains.^4,5^

Sequencing data suggest that the vast majority (∼96%) of rifampicin resistance is associated with mutations in the rifampicin resistance determinant region (RRDR), an 81-base-pair section of the *rpoB* gene spanning­(*MTB*) *rpoB* codons 426 to 452.^6,7^ As a result, current commercial and WHO-endorsed genotypic drug susceptibility tests, such as line-probe assays and Xpert MTB/RIF, focus on detecting rifampicin resistance-related mutations within the RRDR.^6^

However, the landscape of rifampicin resistance continues to evolve with new resistance-related mutations emerging in *rpoB* hotspots outside the current RRDR screening region. Mutations at *rpoB* codon 491, for example, confer rifampicin resistance but remain undetected by current WHO-endorsed genotypic susceptibility tests^6,8,9^ and are routinely misidentified as rifampicin-susceptible by phenotypic drug susceptibility tests.^3,8,10–12^ The *rpoB* I491F mutation, first reported in Eswatini in 2015—where it had been silently transmitted since 2009 and caused a multidrug-resistant outbreak^8^—illustrates this screening gap. While I491F accounts for approximately 0.5% of global rifampicin resistance,^13^ it is far more prevalent in certain regions, accounting for over 50% of rifampicin-resistant cases in Eswatini.^6^ Despite its clinical significance, no current commercial or WHO-endorsed tests screen for rifampicin susceptibility at codon 491.^6,8,9,14^ Moreover, the I491F mutation continues to spread, as indicated by its recent detection in new geographical regions.^9,12,15–17^ In addition, newly identified codon 491 variants, such as I491N and I491M, have also been linked to rifampicin resistance.^18,19^ Susceptibility screening diagnostics are not keeping pace with these evolving resistance-related mutations.

While sequencing has been used for epidemiological discovery and the study of I491 mutations,^3,8,9,15–17,20,21^ the published literature describes only one real-time PCR assay developed for I491F-specific screening.^14^ In this work by André et al., the assay was designed to screen for the I491F mutation by specifically targeting single nucleotide variants (SNVs) at the first nucleotide base of codon 491, *rpoB* nucleotide 1471. Although the André assay’s selective mechanism of action successfully classifies the I491F variant as not susceptible, its specificity prevents its use as is to screen for other resistance-related mutations emerging in the *rpoB* codon 491 hotspot.

In this report, a single-sample screening strategy using asymmetric PCR followed by melt analysis is developed to address the unmet need for comprehensive rifampicin susceptibility screening across all three nucleotides of the *rpoB* codon 491 mutation hotspot. The generalizable approach, termed Single-Sample Melt Analysis for Screening Hotspots (SMASH), is applied here as a proof of concept for screening the *rpoB* codon 491 hotspot. The assay screens for SNVs within *rpoB* nucleotides 1447–1476 using two internal comparators for rifampicin susceptibility in every sample: a single-stranded melt probe and a double-stranded left-helical (L)-DNA. Single-sample classification is achieved by measuring melt temperature (*T*_m_) shifts between two duplexes: the susceptible melt probe hybridized to the asymmetric PCR excess strand and a double-stranded L-DNA with the same *T*_m_ as the susceptible probe-wild-type product duplex. To validate SMASH for I491 screening, we tested its performance across two calibrated real-time PCR instruments (QuantStudio 5 and Rotor-Gene Q) and compared it to the André I491F assay. This evaluation used samples containing synthetic *rpoB* wild-type and three sequence-identified *rpoB* codon 491 variants (I491F/N/M) to validate the assay for SNVs at all three nucleotides (1471–1473) of the *rpoB* codon 491 hotspot^18,19,22^ (Table 1). The SMASH strategy provides a generalizable framework for developing single-sample melt-based assays to screen for clinically significant mutations identified through sequencing.

**Table 1:** Sequences (Written 5′-to-3′) of the Drug-Susceptible Wild-Type *rpoB* and Three Sequence-Identified *rpoB* Codon 491 Variants (I491F/N/M)^a^

Variant	Codon 491 Sequence
Wild-type	ATC
I491F	TTC
I491N	AAC
I491M	ATA

a Each variant has rifampicin resistance-related mutations indicated by an underline.

## Results

Across two real-time PCR instruments, the SMASH assay for I491 successfully classified all *rpoB* codon 491 variants (I491F, I491N, and I491M) as not susceptible and all wild-type *rpoB* samples as susceptible using single-sample melt-based screening (Figures 1A and 2A). Based on Receiver Operating Characteristic (ROC) analysis of the SMASH assay across both the QuantStudio 5 and Rotor-Gene Q sample sets (Table S3), samples were classified as rifampicin-susceptible when the within-sample *T*_m_ difference (between L-DNA *T*_m_ and the probe-product duplex *T*_m_) was less than 0.83 °C (Figures 1A and Figure 2A). Using the QuantStudio 5, average within-sample *T*_m_ differences for wild-type, I491F, I491N, and I491M samples were −0.23 ± 0.21, 2.68 ± 0.16, 2.55 ± 0.14, and 2.75 ± 0.08, respectively (mean ± SD, Figure 1A). Using the Rotor-Gene Q, average within-sample *T*_m_ differences for wild-type, I491F, I491N, and I491M samples were −0.08 ± 0.21, 2.36 ± 0.48, 1.87 ± 0.31, and 2.39 ± 0.48, respectively (mean ± SD, Figure 2A). Visual alignment and near-zero *T*_m_ differences of the susceptible melt duplexes (top left panels of Figures 1B and 2B) were achieved by adjusting probe concentration (Page S5 and Figure S2), though this step was not essential for the within-sample melt comparison strategy. Significant differences were found between wild-type and each of the three variants when the assay was performed in the QuantStudio 5 (Figure 1A) as well as the Rotor-Gene Q (Figure 2A, *p* < 0.0001, one-way ANOVA, Tukey’s post hoc test for multiple comparisons, *n* = 6 trials in triplicate per sample type per instrument). Both PCR systems demonstrated 100% sensitivity and 100% specificity when classifying rifampicin susceptibility (*n* = 6 trials in triplicate per sample type per instrument, Figures 1A and Figure 2A). Successful sample classification is illustrated by 18/18 wild-type *rpoB* samples below the susceptible cutoff and 54/54 variant I491 samples above the susceptible cutoff, in both the QuantStudio 5 (Figure 1A) and Rotor-Gene Q (Figure 2A) sample sets.

**Figure 1:**
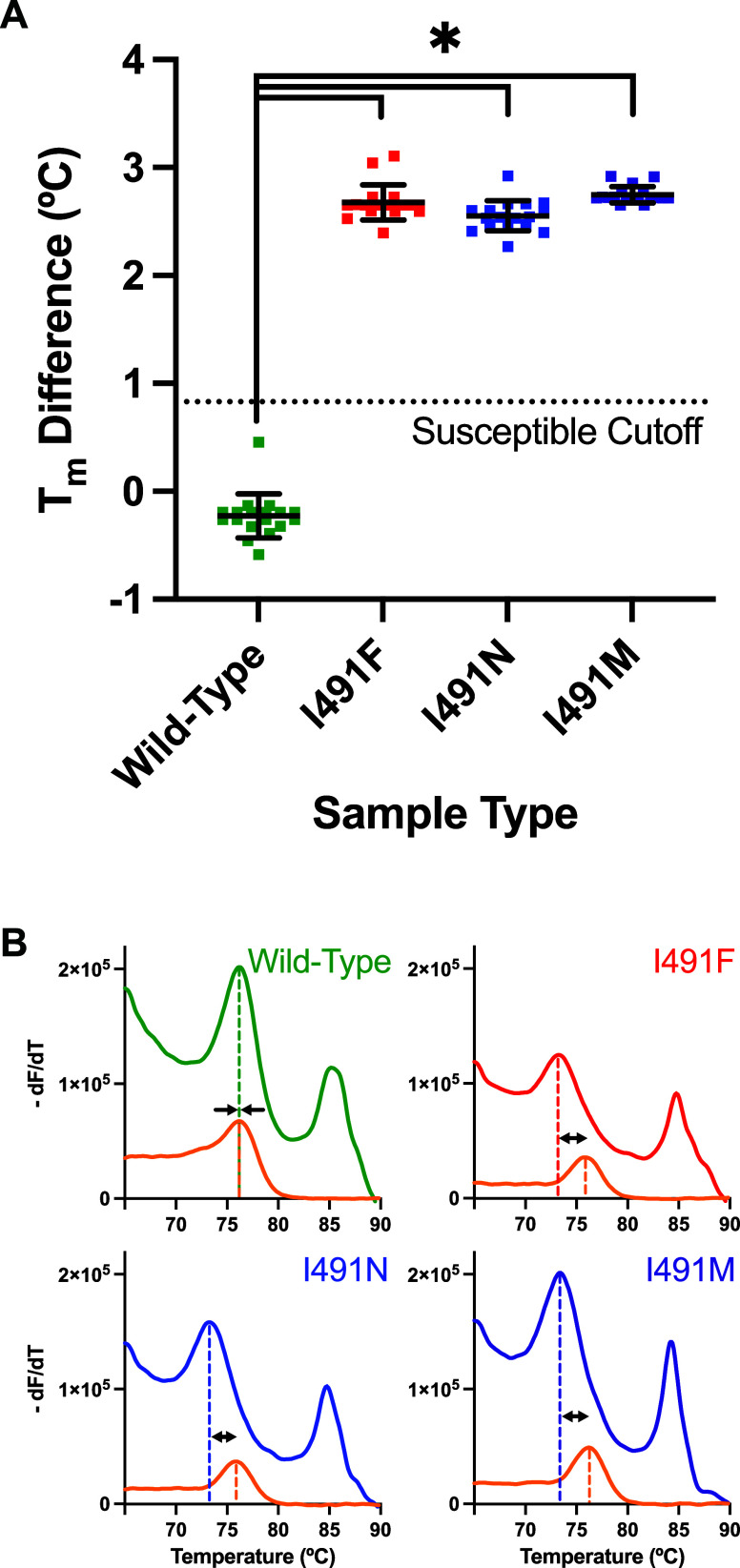
Performance of the SMASH assay using the QuantStudio 5 real-time PCR instrument. (A) Within-sample *T*_m_ differences of SMASH samples across wild-type (green) and variants I491F (red), I491N (blue), and I491M (purple). Average *T*_m_ difference ± standard deviation per sample type is indicated across *n* = 6 trials in triplicate per sample type. (B) Within-sample *T*_m_ comparison for four representative samples containing susceptible L-DNA (orange) and susceptible melt probe duplexed to wild-type or variant asymmetric PCR product (green, red, blue, purple). Dashed vertical lines indicate *T*_m_s within each sample for L-DNA (orange) and the probe-product duplex (green, red, blue, and purple).

**Figure 2:**
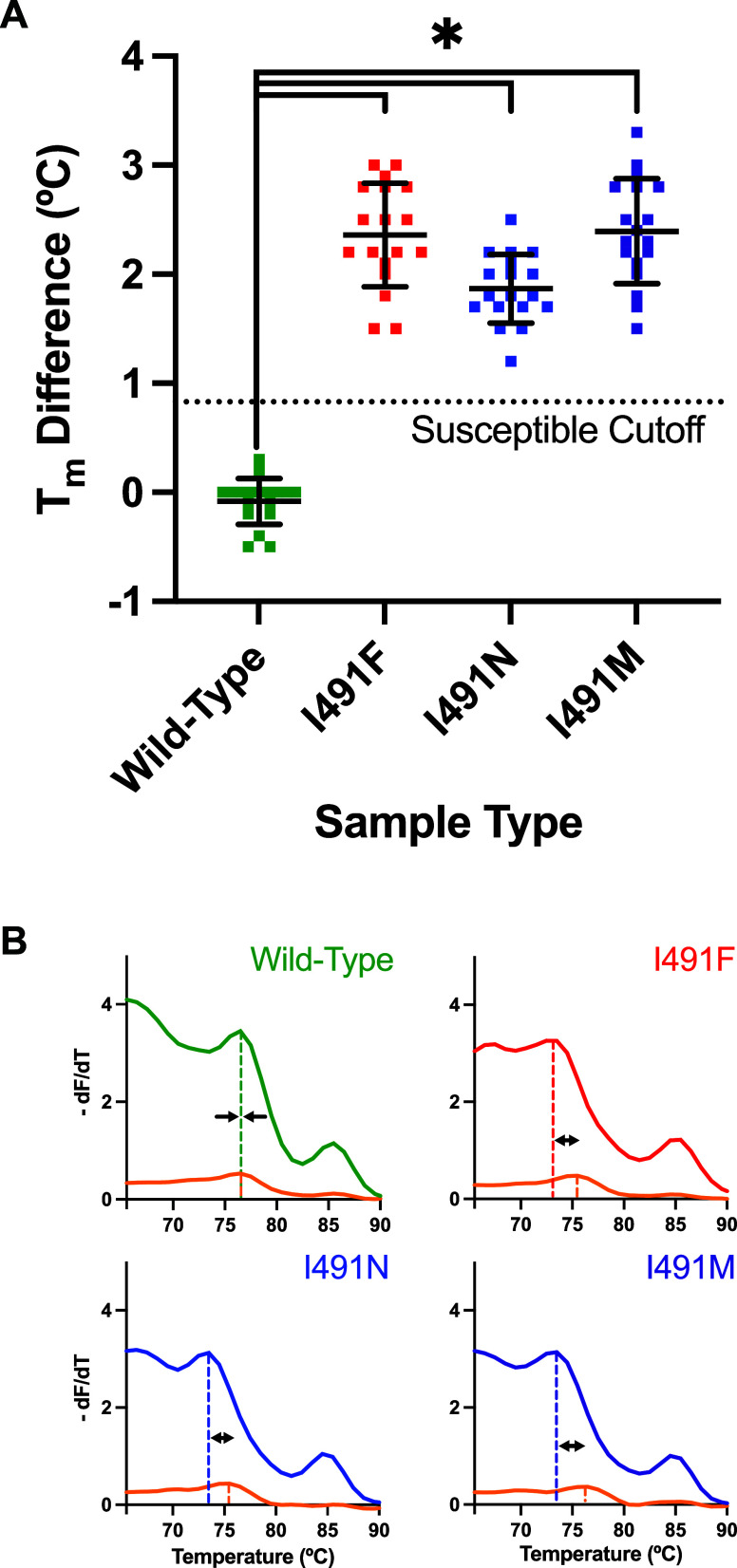
Performance of the SMASH assay using the Rotor-Gene Q real-time PCR instrument. (A) Within-sample *T*_m_ differences of SMASH samples across wild-type (green) and variants I491F (red), I491N (blue), and I491M (purple). Average *T*_m_ difference ± standard deviation per sample type is indicated across *n* = 6 trials in triplicate per sample type. (B) Within-sample *T*_m_ comparison for four representative samples containing susceptible L-DNA (orange) and susceptible melt probe duplexed to wild-type or variant asymmetric PCR product (green, red, blue, purple). Dashed vertical lines indicate *T*_m_s within each sample for L-DNA (orange) and the probe-product duplex (green, red, blue, and purple).

The performance of the SMASH assay for I491 was compared to the reference André I491F assay using the same *rpoB* test system in the QuantStudio 5. While the André I491F assay confirmed successful classification of all wild-type *rpoB* and I491F variant samples, the expansion of this test to I491N and I491M variant samples resulted in misclassification (Figure 3A). Per André et al.,^14^ samples were classified as rifampicin-susceptible using a *T*_m_ difference less than 5.24 °C from the global average wild-type melt temperature from the sample set (*n* = 3 trials in triplicate per sample type). As supported by prior literature,^14^ melt characteristics differed between wild-type *rpoB* and variant I491F (Figure 3B). Variants I491N and I491M, however, had nearly identical melt characteristics to wild-type *rpoB* despite exhibiting SNVs (Figure 3B). Average *T*_m_ differences for wild-type, I491F, I491N, and I491M were 0.00 ± 0.14, 5.90 ± 0.05, −0.08 ± 0.16, and 0.07 ± 0.07, respectively (mean ± SD). Using melt difference comparison, there was a significant difference between wild-type and I491F (*p* < 0.0001) but no significant difference between wild-type and I491N or wild-type and I491M (*p* > 0.05, one-way ANOVA, Tukey’s post hoc test for multiple comparisons, 9 replicates per sample type, Figure 3A). Successful sample classification is illustrated by 9/9 wild-type *rpoB* samples below the susceptible cutoff and 9/9 variant I491F samples above the susceptible cutoff (Figure 3A, *n* = 3 trials in triplicate per sample type). Misclassification is illustrated by 9/9 I491N samples and 9/9 I491M samples below the susceptible cutoff (*n* = 3 trials in triplicate per sample type, Figure 3A).

**Figure 3:**
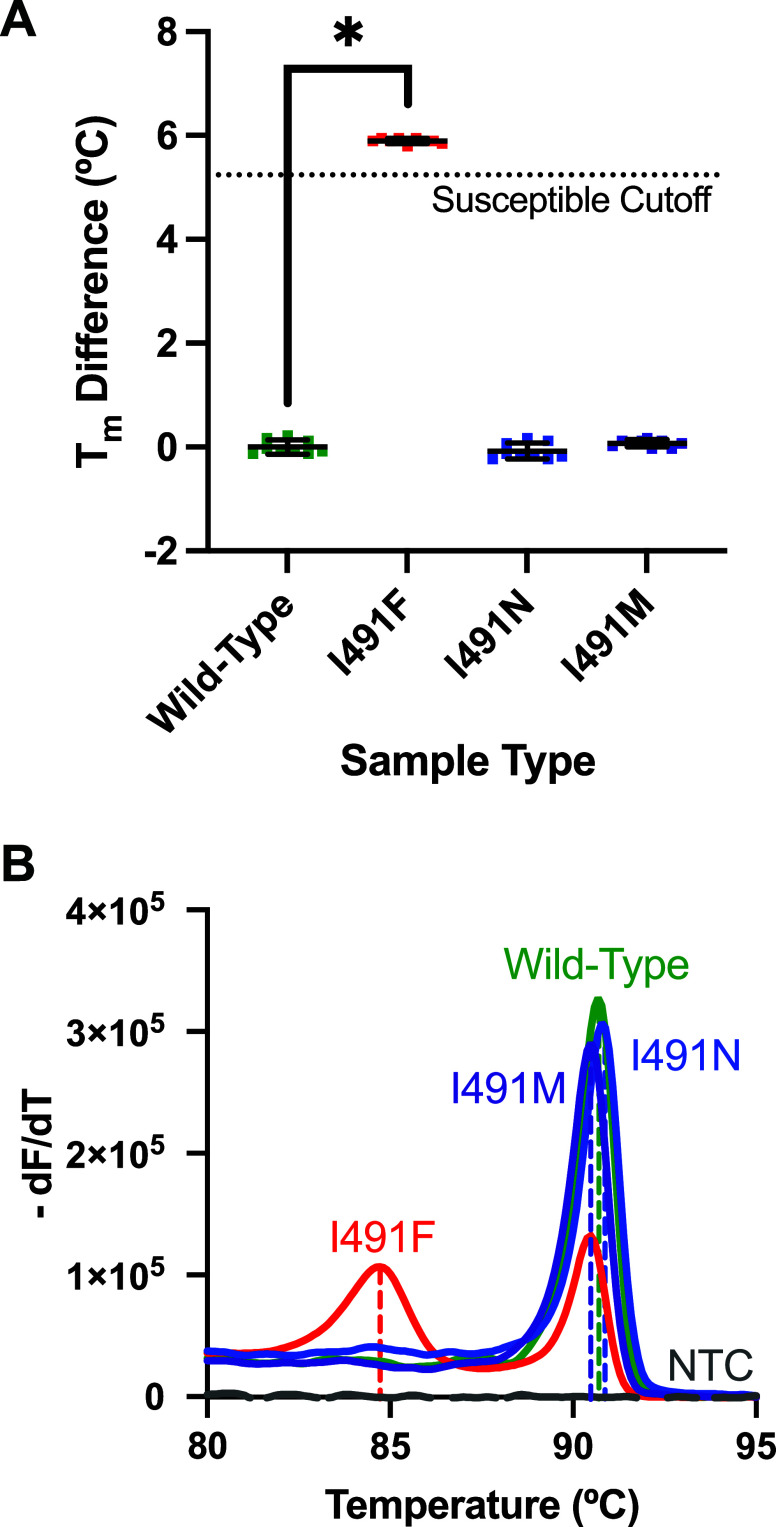
Performance of the André I491F assay using the QuantStudio 5 real-time PCR instrument. (A) *T*_m_ differences between each sample’s PCR product *T*_m_ and the sample set’s global average wild-type *T*_m_ across wild-type (green) and variants I491F (red), I491N (blue), and I491M (purple). Average *T*_m_ difference ± standard deviation per sample type is indicated across *n* = 3 trials in triplicate per sample type. (B) Multisample *T*_m_ comparison for five representative samples plotted together containing either wild-type (green), one of three variants (red, blue, purple), or no template control (NTC, gray). Dashed vertical lines indicate the *T*_m_ values for each sample.

## Discussion

The incorporation of internal comparators in the SMASH assay enabled each unknown sample to be successfully classified as susceptible or not without the need for a comparison to other samples. This single-sample melt-based approach effectively screened for rifampicin susceptibility among wild-type *rpoB* and three sequence-identified *rpoB* variants: I491F, I491N, and I491M. The assay achieved 100% sensitivity and 100% specificity in classifying rifampicin susceptibility of synthetic unknown samples (*n* = 6 trials in triplicate per sample type per instrument) across multiple real-time PCR platforms (QuantStudio 5 and Rotor-Gene Q, Figures 1A and 2A). Its consistent high performance across two platforms highlights the method’s robustness and adaptability, ensuring broader TB screening access using existing instruments—without the need for assay-specific equipment investments.

Notably, a single susceptibility cutoff was applied uniformly across both instruments (Table S3), simplifying the SMASH strategy by eliminating instrument-specific optimizations. This reinforces SMASH’s potential for implementation on any real-time PCR system with built-in melt analysis capabilities, offering a broadly applicable, instrument-agnostic solution for rapid rifampicin susceptibility screening.

Prior *rpoB* I491 screening efforts relied on either resource-intensive sequencing methods^3,8,9,15–17,20,21^ covering the entire *rpoB* gene or highly specific real-time melt analysis targeting *rpoB* nucleotide 1471, the single base location of I491F.^14^ In our report, the melt-based I491F-specific André assay was revisited with an expanded variant set, incorporating SNVs at all three positions of codon 491 using rifampicin resistance-related variants I491F, I491N, and I491M.^18,19,22^ While the André I491F assay successfully classified I491F as not susceptible, its selective mechanism of action failed to correctly classify other resistance-related mutations within the *rpoB* codon 491 hotspot (Figure 3A). Comprehensive screening of all three nucleotides in codon 491 would require two additional iterations of the André assay, each targeting one of the remaining *rpoB* nucleotides (1472 and 1473). This would necessitate three separate samples to screen for SNVs at all three positions of codon 491—a workflow that adds additional complexity into the TB clinical treatment algorithm.

### Key Factors Driving SMASH Assay Success

The success of our single-sample melt-based assay is driven by five key features: (1) including a susceptible melt probe in every sample, (2) selecting the melt probe sequence to maximize mutation-induced melt shifts, (3) incorporating a susceptible L-DNA comparator for internal *T*_m_ calibration, (4) adjusting melt probe concentration to align *T*_m_s of the two susceptible duplexes (double-stranded L-DNA and the probe–wild-type product duplex), and (5) collecting melt data from L-DNA and D-DNA on separate optical channels.

#### Inclusion of a Susceptible Melt Probe

The susceptible melt probe was incorporated into every sample and designed as the reverse complement of the drug-susceptible *rpoB* sequence. This ensured that the melt probe could hybridize with the excess asymmetric PCR product strand regardless of whether the unknown sample contained a wild-type *rpoB* sequence.

#### Optimized Melt Probe Sequence for Maximized Melt Shifts

The susceptible melt probe sequence was strategically selected to maximize the melt shift between its duplex with a wild-type strand and its duplex with an I491F strand (see Page S3 and Figure S1). Consistent with prior literature,^23,24^ increasing the distance of the SNV (I491F at *rpoB* nucleotide 1471) from the probe’s 5′ end increased the melt shift between the probe-I491F and the probe-wild-type duplexes, likely due to greater duplex destabilization caused by the mismatch^23,24^ (Figure S1). This optimized melt difference facilitated clear discrimination between the I491F samples and the susceptible L-DNA melt behavior (Figures 1A and 2A). Similarly, mismatched probe-product duplexes from I491N and I491M samples exhibited distinct melt differences from susceptible profiles (Figures 1A and 2A).

Since the melt probe sequence defines the SMASH assay’s screening region, this 30-base probe span determines which existing or potentially emerging mutations can be identified. The assay may detect other SNVs, beyond the I491F/M/N variants validated here, that cluster within this 30-base region around the codon 491 hotspot, provided a mutation induces a sufficiently distinguishable melt shift. For example, the recently identified I491V and I491L variants, which may contribute to rifampicin resistance,^18,19^ demonstrate a pattern of recurrent mutations in this region. This highlights a use case where SMASH’s broad screening capability can be capitalized on. By leveraging the tendency of resistance-related mutations to cluster, the SMASH strategy has the potential to detect both established, sequence-identified mutations—which are expected to become more prevalent—and emerging mutations that may arise over time.

#### Inclusion of a Susceptible L-DNA Comparator for Internal Calibration

The double-stranded L-DNA comparator was designed to match the duplex sequence of the susceptible probe hybridized to a wild-type asymmetric PCR product, making L-DNA an effective melt comparator for susceptibility classification. SMASH incorporates L-DNA in every sample to achieve a reagent-based calibration,^25^ enabling single-sample classification without requiring multisample comparisons, while also correcting for potential assay variations across reaction wells or plates. Similar L-DNA-based strategies have been successfully employed in prior work, including a 56-base double-stranded L-DNA melt comparator for isoniazid susceptibility.^25^

Consistent with previous research,^25–28^ the melt characteristics of susceptible-sequence D-DNA (melt probe bound to wild-type asymmetric PCR product) and L-DNA (double-stranded melt comparator) were nearly identical (top left panels in Figures 1B and 2B). SMASH melt analysis compared the sequence-specific *T*_m_ of the L-DNA comparator to that of the probe-product duplex. A *T*_m_ shift in the probe-product duplex relative to the susceptible L-DNA comparator indicated the presence of a SNV in the unknown sample (*rpoB* I491 variants in Figures 1 and 2). This internal melt comparison strategy facilitated a high-performance single-sample classification.

#### Adjusting Melt Probe Concentration for Visual Melt Alignment

Single-sample classification was further enhanced by adjusting the melt probe concentration to align the melt temperatures of the two susceptible duplexes: double-stranded L-DNA and the susceptible melt probe bound to the wild-type asymmetric PCR product.

While achieving a near zero *T*_m_ difference simplified visual interpretation of melt curves as susceptible or not (Figures 1B and 2B), matching was not strictly necessary. The *T*_m_ difference remains constant for a fixed probe concentration, allowing for reliable sample classification based on this invariant offset.

To optimize melt matching, the susceptible melt probe concentration was adjusted so that *T*_m_ of its duplex with the wild-type asymmetric PCR product aligned with that of the double-stranded L-DNA comparator (see Page S5 and Figure S2). Previous studies have demonstrated that total DNA concentration and strand ratio affect the melt temperature,^29–32^ a principle applied in our previous L-DNA-based work where the susceptible L-DNA concentration was adjusted so that L-DNA melt characteristics aligned with that of the wild-type PCR amplicon.^25^ In the present study, the susceptible melt probe concentration was reduced to ensure that *T*_m_ of its duplex to the wild-type asymmetric PCR product matched L-DNA *T*_m_ (Figure S2). This empirical melt match between susceptible duplexes (top left panels in Figures 1B and 2B) only needed to be completed one time in test development and contributed to successful implementation of L-DNA for rifampicin susceptibility screening.

#### Collecting L-DNA and D-DNA Melt Data on Separate Optical Channels

To facilitate single-sample classification and discriminate between L-DNA and D-DNA melting behavior within one reaction, the SMASH assay collects L-DNA fluorescent melt data on a separate optical channel from D-DNA. This distinction was necessary because all readily available intercalating dyes do not discriminate between enantiomeric DNA.^25^ In our related L-DNA-based isoniazid susceptibility assay, intercalating crosstalk was overcome by labeling double-stranded L-DNA with a Texas Red fluorophore and a quencher. This enabled L-DNA melt monitoring in a separate optical channel from the green intercalating dye used for the D-DNA PCR products.^25^ However, the fluorescence resonance energy transfer (FRET) mechanism between the fluorophore and quencher during melting causes a rise in donor fluorescence,^33^ resulting in a negative melt curve. Since many real-time PCR instruments are unable to determine *T*_m_ from such curves, this design required postprocessing steps to accurately determine L-DNA *T*_m_, adding extra steps to the workflow.

To overcome this challenge, the present study implemented intercalated FRET (iFRET)^33^ to capture L-DNA melt data. Probe-product duplex melting was monitored on the green optical channel via SYBR Green I intercalating dye, while L-DNA melting was monitored on the orange optical channel using iFRET, with SYBR Green I as the FRET donor and Texas Red (labeled on the L-DNA forward strand) as the FRET acceptor. Unlike traditional fluorophore-quencher FRET, iFRET results in a drop in acceptor fluorescence during melting,^33^ generating a positive melt curve that can be automatically analyzed by built-in PCR instrument software to calculate *T*_m_. This eliminates the need for postprocessing and simplifies the assay workflow.

Beyond workflow efficiency, iFRET offers cost and accuracy advantages. iFRET is more cost-effective than traditional fluorophore-quencher FRET designs since it requires only a single fluorophore label on L-DNA. Furthermore, iFRET preserves melt measurement accuracy; *T*_m_ values from iFRET were equivalent to those obtained via intercalating dye measurements, as confirmed by L-DNA melting in no template control (NTC) samples (Figure S6).

For iFRET to function, the L-DNA fluorophore must have a sufficient spectral overlap with the intercalating dye. Additionally, the real-time PCR instrument must support excitation in one optical channel and emission in another. Common real-time PCR instruments such as the LightCycler 480,^34^ QuantStudio 3,^35^ QuantStudio 5,^35^ and Rotor-Gene Q^36^ meet this requirement.

### Extending the Strategy to Other Hotspots

The SMASH screening strategy presented in this report is highly generalizable and can be adapted to other mutation hotspots. Its implementation depends on sequencing efforts to identify mutations in emerging hotspots and on clinical and epidemiological evidence linking these sequence-identified variants to pathogenic impact. Once such associations are established, hotspot-specific SMASH can be implemented in four steps. First, PCR primers are designed to amplify the target screening region. Second, a susceptible melt probe is selected to define the exact nucleotide screening span and optimized to maximize mutation-induced melt shifts. Third, a left-handed L-DNA sequence is synthesized to mimic the susceptible duplex formed by the melt probe hybridized to the wild-type asymmetric PCR product. The screening region length is constrained by two factors: the synthesis limitations of L-DNA (biomers.net) and the decreasing detectability of SNV-induced melt temperature shifts as sequence length increases.^37^ Fourth, the melt probe concentration is adjusted, if desired, to align the melt temperatures of the probe-wild-type product duplex with the susceptible L-DNA. While this step simplifies visual interpretation of melt curves as susceptible or not, it is not strictly necessary. The *T*_m_ difference remains constant for a fixed probe concentration, ensuring reliable sample classification, even without matching the melt curves.

### Resource-Constrained Alternative Designs

Less than 1% of the SMASH assay’s reagent cost is attributed to L-DNA. The L-DNA primarily serves as a susceptible melt comparator in the SMASH assay for I491. Our previous work also suggests that L-DNA functions as an internal melt calibrator, correcting for within-assay variability (such as well-to-well or plate-to-plate differences) that could otherwise reduce classification performance.^25^ This within-sample correction compensates for systematic hybridization changes caused by sample preparation errors or other sources of variability, such as culture media carryover, extraction errors, kit-to-kit master mix differences, or sample-to-sample salt concentration variability resulting from reagent pipetting errors.^38^ The inclusion of L-DNA for reagent-based calibration suggests that even though the SMASH assay was tested on two state-of-the-art calibrated instruments, highly calibrated equipment may not be essential for successful SMASH assay implementation. Instead, the calibration function is provided by L-DNA itself. While L-DNA-based calibration effectively corrects for within-assay variability, it is important to note that within-sample *T*_m_ differences may still vary slightly between instruments, as demonstrated in this study across two different platforms.

Despite the benefits of incorporating an L-DNA comparator in every sample, certain use-cases for I491 screening may arise where iFRET-capable instruments are unavailable or other L-DNA requirements are not met. The SMASH I491 assay developed in this study can be adapted to be L-DNA-independent while still maintaining high performance (see Page S9 and Figure S7). This alternative approach demonstrates how the SMASH I491 screening assay can be analyzed using a traditional melt probe method with multisample comparison.^39–41^ When L-DNA melt data are excluded from classification analysis on highly calibrated, real-time PCR instruments (QuantStudio 5 and Rotor-Gene Q), multisample *T*_m_ comparison—similar to the strategy used to analyze the André assay sample set—still consistently achieved 100% sensitivity and 100% specificity in classifying rifampicin susceptibility in unknown synthetic samples (*n* = 6 trials in triplicate per sample type per instrument, Figure S7). In this case, the *T*_m_ difference was calculated as the difference between each sample’s probe-product duplex *T*_m_ and the sample set’s global average *T*_m_ of the probe bound to wild-type product. However, this L-DNA-independent approach would rely on historical melt data for classification, eliminating single-sample classification capabilities, and may be more prone to within-assay variability, potentially compromising accuracy—whereas L-DNA could correct for such variability as an internal melt standard.

### Future Directions

This study establishes SMASH as a promising single-sample melt-based strategy for mutation screening at *rpoB* codon 491, with a strong proof of concept using a synthetic *rpoB* system. However, validation in clinical samples is still needed.

The strong selective pressure exerted by anti-TB drugs drives the emergence and spread of drug-resistant *MTB* strains. As resistance-related mutations accumulate, their prevalence and clinical significance are expected to increase, making early and accurate detection critical.

*MTB* resistance mutations often cluster in well-characterized regions, such as the RRDR of *rpoB*, which accounts for approximately 96% of rifampicin resistance,^6,7^ and the quinolone resistance-determining region (QRDR) of *gyrA* and *gyrB*, responsible for 50–90% and 7% of fluoroquinolone resistance, respectively.^42^ These clustering patterns underscore the need for targeted hotspot screening.

SMASH’s conservative design, which scans regions surrounding known hotspots, enhances its ability to detect both established resistance mutations and newly emerging variants clustering within these critical regions. By leveraging the natural aggregation of resistance mutations, SMASH serves as both a powerful screening tool and an anticipatory strategy for resistance surveillance. Expanding SMASH to additional *MTB* regions with well-documented hotspot aggregation could further improve resistance monitoring.

Currently, SMASH is applicable when target mutations serve as unique classifiers of disease susceptibility. A key future advancement would be multiplexing SMASH to simultaneously screen multiple hotspots critical to clinical outcomes. For example, a multiplexed SMASH assay could assess rifampicin susceptibility by screening both the RRDR and *rpoB* codon 491 in a single reaction, improving the diagnostic efficiency.

Beyond TB, SMASH’s adaptability can facilitate the detection of clinically significant mutations in other genetic hotspots linked to disease susceptibility.^43–45^ For instance, *TP53* mutations are associated with malignant progression and chemoresistance,^46,47^ while *gyrA* mutations contribute to drug resistance in bacterial pathogens, such as *corynebacteria* ^48^ and .^49^ By facilitating the targeted screening of these regions, SMASH has the potential to deepen our understanding of disease etiology, refine therapeutic and diagnostic strategies, and support the advancement of personalized medicine.

## Conclusion

This study establishes SMASH as a new single-sample strategy for screening sequence-identified mutations with clinical significance. SMASH was successfully applied to rifampicin susceptibility screening at the commercially unaddressed *rpoB* 491 mutation hotspot, achieving 100% sensitivity and 100% specificity. Beyond TB drug resistance, SMASH offers a generalizable framework adaptable to other disease susceptibility applications, with the potential to improve diagnostic accuracy and inform clinical decision-making.

## Materials and Methods

### Single-Sample Melt Analysis for Screening Hotspots (SMASH) Applied to the Hotspot at *rpoB* Codon 491

The single-sample melt analysis test was developed by using the sequence of the drug-susceptible TB *rpoB* gene. A single primer set was designed to cover rifampicin resistance-related mutations at the *rpoB* codon 491 emerging hotspot^18,19,22^ and its neighboring bases. SMASH uses asymmetric PCR followed by melt analysis, where asymmetric PCR generates both single-stranded asymmetric products and full-length amplicons.

A drug-susceptible melt probe^50^ was synthesized as a reverse complement sequence to the known drug-susceptible *rpoB* sequence. The 30-base melt probe sequence was identical to the corresponding region of the excess asymmetric PCR product strand between the primers. The susceptible melt probe had a 3′ C3 spacer to prevent extension during PCR. The 30-base susceptible melt probe formed a duplex with the 66-base asymmetric PCR product, regardless of whether the PCR product had a susceptible sequence. If a SNV was present in the PCR product strand, the probe-product duplex had a nucleotide mismatch that destabilized the duplex^23,24^ and caused a melt mismatch with the susceptible L-DNA’s *T*_m_. Strategic selection of the melt probe sequence is detailed in Page S3 and Figure S1.

Similar to prior L-DNA-based screening for isoniazid susceptibility,^25^ this assay added double-stranded L-DNA to every sample to serve as a standard melt comparator for rifampicin susceptibility. The L-DNA drug-susceptible comparator was synthesized using left-helical enantiomeric DNA bases (i.e., L-DNA)^51^ with an identical sequence to the known drug-susceptible *rpoB* sequence. The 30-base L-DNA forward strand was synthesized with the same length and sequence as the drug-susceptible melt probe. The forward L-DNA strand had a 5′ Texas Red end-label. The reverse complement L-DNA strand was unlabeled.

The assay was performed on a test system of synthetic *rpoB* wild-type and three variant sample types. Single-stranded PCR targets were synthesized with D-DNA sequences of drug-susceptible wild-type *rpoB* (H37Rv: Rv0667:759807–763325) and three clinically relevant drug-resistant *rpoB* mutants (Table 1). Each of the three selected mutants included SNVs at one of the three nucleotides of *rpoB* codon 491,^18,19,22^ offering robust SMASH assay validation for rifampicin susceptibility at *rpoB* codon 491. Detailed information about the DNA oligonucleotide sequences used in these studies is shown in Table S1. All DNA oligonucleotides employed for development and testing were synthesized by Integrated DNA Technologies (Coralville, Iowa, USA) or biomers.net (Ulm, Baden-Württemberg, Germany).

The SMASH assay was performed using two real-time PCR thermal cyclers, the QuantStudio 5 instrument (Thermo Fisher Scientific #A28137) with a 96-well plate sample setup, and the Rotor-Gene Q 5plex Platform (Qiagen #9001570) with a spinning rotor sample setup. Assay performance was compared across two real-time PCR platforms to demonstrate its versatility and adaptability across systems, ensuring broader TB screening access using existing instruments rather than requiring assay-specific equipment investments. All reactions had a 20 μL final volume containing 1× SensiFAST Probe No-ROX Kit (Bioline #BIO-86005), 1× SYBR Green I (Sigma-Aldrich #S9430), 0.25 μM *rpoB*-specific forward primer (SMASH_FWDpri), 0.025 μM *rpoB*-specific reverse primer (SMASH_REVpri), 3.7625 × 10^12^ copies of C3-blocked susceptible melt probe (SMASH_SusMeltProbeC3), 4 × 10^11^ copies of Texas Red-labeled forward strand L-DNA (SMASH_FWDsusLDNAtxr), and 4 × 10^11^ copies of unlabeled reverse strand L-DNA (SMASH_REVCOMPsusLDNA). Each target sample contained a final concentration of a wild-type (TARGETrpoB_WT) or mutant (TARGETrpoB_I491F, TARGETrpoB_I491N, TARGETrpoB_I491M) single-stranded DNA target at 2 × 10^6^ copies per reaction. In preliminary tests, the susceptible melt probe concentration was reduced per reaction to align *T*_m_ of the duplexed susceptible melt probe with the wild-type asymmetric PCR product to that of the double-stranded L-DNA (Page S5 and Figure S2). This concentration-based strategy, similarly employed in prior L-DNA-based work,^25^ enabled single-sample testing by ensuring that the susceptible L-DNA and the probe-product duplex had nearly identical melt characteristics when the sequences matched (wild-type *rpoB*) but differed if there was a sequence mismatch (*rpoB* I491 variants).

QuantStudio 5 PCR reactions were initiated with a 95 °C hold for 2 min followed by 40 cycles of 95 °C for 5 s and 57 °C for 20 s. A high-resolution melt was performed immediately following PCR by annealing from 95 to 50 °C at 0.1 °C/s followed by melting from 65 to 90 °C at 0.025 °C/s (continuous acquisition mode). Double-stranded DNA PCR product fluorescence was measured during PCR and during melting using SYBR Green I on the green optical channel (excitation 470 ± 15/emission 520 ± 15). Double-stranded L-DNA fluorescence was monitored on the orange optical channel (emission 623 ± 14) during the melt reaction using iFRET^33^ with SYBR Green I as the FRET donor and Texas Red on the L-DNA forward strand as the FRET acceptor.

Rotor-Gene Q PCR reactions followed an identical reaction setup and PCR cycling conditions to QuantStudio 5. Melt analysis data acquisition rates and optical channel specifications varied between instruments due to inherent differences in their design. The Rotor-Gene Q’s high-resolution melt was performed immediately following PCR by annealing from 95 to 50 °C at 0.1 °C/step followed by melting from 65 to 95 °C at 1 °C/step with 5 s hold per step. The Rotor-Gene Q’s SYBR Green I optical channel was specified by excitation 470 ± 10/emission 510 ± 5, while the iFRET acceptor orange optical channel was specified by excitation 470 ± 10/emission 610 ± 5.

PCR quantification cycle (C_q_) was determined with the QuantStudio 5 Design and Analysis Software and the Rotor-Gene Q Series Software. Representative PCR amplification curves of samples for the QuantStudio 5 and Rotor-Gene Q are included in Figures S3 and S4, respectively. *T*_m_ was calculated with the proprietary QuantStudio 5 Design and Analysis Software based on the first derivative of fluorescence with respect to temperature. Within-sample melt difference and statistical significance were performed independently for the QuantStudio 5 and Rotor-Gene Q data sets. A within-sample melt difference was calculated between *T*_m_ of each sample’s probe-product duplex and *T*_m_ of each sample’s L-DNA. Significance was evaluated using the within-sample melt difference comparison (one-way ANOVA, Tukey’s post hoc test for multiple comparisons, significance level of α=0.95) across I491F, I491N, and I491M as compared to wild-type (*n* = 6 trials in triplicate per sample type per instrument). Since true positives were known, the SMASH assay was assessed for its sensitivity and specificity using ROC analysis of within-sample *T*_m_ difference metrics (Wilson/Brown method, Table S3). Across both the QuantStudio 5 and Rotor-Gene Q sample sets, a *T*_m_ difference cutoff point was selected by prioritizing maximized specificity (to decrease the false positive rate, i.e., decrease the misdiagnosis of variant samples as drug-susceptible), followed by maximized sensitivity, when classifying each test sample as drug-susceptible or not. All samples were classified as rifampicin-susceptible using a within-sample *T*_m_ difference less than 0.83 °C for the QuantStudio 5 and Rotor-Gene Q sample sets (*n* = 6 trials in triplicate per sample type per instrument). All statistics were performed in GraphPad Prism version 10.0.

### André I491F Assay

The André et al. assay for I491F-specific detection^14^ was replicated as closely as possible to the original published method to compare the performance of the developed SMASH assay with existing real-time PCR I491F screening capabilities. The assay’s multiplex allele-specific PCR strategy used a combination of three primers to amplify allele-specific fragments distinguished by their size. According to prior work, rifampicin-susceptible wild-type samples were expected to form 301 base-pair PCR amplicons, while I491F samples were expected to form 70 base-pair PCR amplicons. The André I491F assay was performed using the same synthetic targets (Table 1) and QuantStudio 5 instrument as the SMASH assay for comparison. All reactions had a 25 μL final volume containing 1× of SensiFAST Probe No-ROX Kit, 1× SYBR Green I, and 0.5 μM of each *rpoB*-specific MAS-PCR primer (Andre_FWDpri_outer, Andre_FWDpri_overlapping1471, Andre_REVpri). Each target sample contained a final concentration of a wild-type (TARGETrpoB_WT) or mutant (TARGETrpoB_I491F, TARGETrpoB_I491N, TARGETrpoB_I491M) single-stranded DNA target at 5 × 10^6^ copies per reaction. Detailed information on the DNA oligonucleotide sequences used in these studies is shown in Table S1. PCR reactions were initiated with a 95 °C hold for 2 min followed by 35 cycles of 95 °C for 5 s and 56 °C for 20 s. A high-resolution melt was performed immediately following PCR by melting from 65 to 97 °C at 10 readings per °C (dissociation acquisition mode). Double-stranded DNA PCR product fluorescence was measured during PCR and during melting using SYBR Green I on the green optical channel (excitation 470 ± 15/emission 520 ± 15). Protocol deviations from the prior work^14^ included the type of real-time PCR instrument, the type of PCR master mix, melt cycling conditions, the use of synthetic targets instead of clinical samples, and expansion to include variants I491N and I491M in addition to I491F.

QuantStudio 5 Design and Analysis Software determined PCR *C*_q_ and *T*_m_ (based on the first derivative of fluorescence with respect to temperature). Representative PCR amplification curves of samples are included in Figure S5. Although André et al. did not explicitly state how to determine melt difference criteria,^14^ the present study calculated melt difference, or *T*_m_ difference, between *T*_m_ of each sample’s PCR product and *T*_m_ of the global average wild-type melt temperature from the sample set (*n* = 3 trials in triplicate per sample type). Significance was evaluated using melt difference comparison (one-way ANOVA, Tukey’s post hoc test for multiple comparisons, significance level of α=0.95) across I491F, I491N, and I491M as compared to wild-type (*n* = 3 trials in triplicate per sample type). Per André et al.,^14^ samples were classified as rifampicin-susceptible using a *T*_m_ difference less than 5.24 °C (*n* = 3 trials in triplicate per sample type). All statistics were performed in GraphPad Prism version 10.0.

## Supplementary Material



## Notes

Additional experimental details, methods, and materials, including oligonucleotide sequences for this work; experimental characterizations of the susceptible melt probe’s sequence and reaction concentration; representative PCR amplification curves of SMASH assay samples tested using QuantStudio 5 and Rotor-Gene Q; representative PCR amplification curves of André I491F assay samples tested using the QuantStudio 5; SMASH assay melt curves of NTC samples using iFRET versus intercalation alone; ROC analysis for sensitivity and specificity of the SMASH assay; and an alternative classification strategy and associated ROC analysis of the SMASH sample sets using multisample comparison (PDF)
