# Supplementary material for: Single-Sample Melt-Based Screening for Rifampicin Susceptibility in the Emerging Mutation Hotspot at rpoB Codon 491
Source: ACS Infect Dis. 2025 Jun 17;11(7):1934–43. doi: 10.1021/acsinfecdis.5c00150 (PMC12261315; doi:10.1021/acsinfecdis.5c00150)
Supplement: Supplementary file 1 [file id5c00150_si_001.pdf]

Supporting Information

Single-sample melt-based screening for rifampicin susceptibility in the emerging mutation hotspot at *rpoB* codon 491

Nicole A. Malofsky[1], Swayashreyee B. Dhungel[1], Megan E. Pask[1], Frederick R. Haselton[1*]

[1] Department of Biomedical Engineering, Vanderbilt University, Nashville, TN, 37235, United States

* To whom correspondence should be addressed. Tel: (615)-322-6622; Fax: (615)-343-7919; Email: Rick.Haselton@vanderbilt.edu

Table of Contents

Oligonucleotides S2
Selecting Susceptible Melt Probe Sequence S3-S4
Tuning Susceptible Melt Probe Concentration S5
PCR Results S6
Comparing SMASH Assay Melt Curves of NTC Samples Using iFRET Versus Intercalation Alone S7
ROC Analysis Using Within-Sample T_m Differences on SMASH Sample Sets S8
Classification and ROC Analysis Using Multi-Sample Comparison on SMASH Sample Sets S9-S11
References for Supporting Information S12

Oligonucleotides

Table S1. Oligonucleotide sequences designed for the SMASH assay applied to *rpoB* codon 491 and the André I491F assay. DNA is denoted as D-DNA or L-DNA. Nucleotides in *rpoB* codon 491 are underlined. Single-base changes of interest in mutants are indicated in red.

ID	DNA Type	Description	Nucleotide Base Change	Sequence (5' → 3')
**SMASH_FWDpri**	D-DNA	SMASH assay *rpoB* forward primer (66 base-pair amplicon)	N/A	GATGTGCCCGATCGAAACC
**SMASH_REVpri**	D-DNA	SMASH assay *rpoB* reverse primer (66 base-pair amplicon)	N/A	GCGTACACCGACAGCGA
**SMASH_SusMeltProbeC3**	D-DNA	SMASH assay rifampicin-susceptible melt probe with 3' C3 spacer to block PCR extension	N/A	GCCGATCAGACCGATGTTGGGCCCCTCAGG/3SpC3/
**SMASH_FWDsusLDNAtxr**	L-DNA	SMASH assay rifampicin-susceptible L-DNA comparator forward strand with 5' Texas Red-X	N/A	Texas Red / GCCGATCAGACCGATGTTGGGCCCCTCAGG
**SMASH_REVCOMPsusLDNA**	L-DNA	SMASH assay rifampicin-susceptible L-DNA comparator reverse complement strand with no modification	N/A	CCTGAGGGGCCCAACATCGGTCTGATCGGC
**Andre_FWDpri_outer**	D-DNA	André I491F assay forward primer (301 base-pair amplicon)	N/A	TGGAGTACGTGCCCTCGTC
**Andre_FWDpri_overlapping1471**	D-DNA	André I491F assay forward primer including nucleotide 1471 of *rpoB* codon 471 (70 base-pair amplicon)	N/A	GGCCCAACATCGGTCTGA
**Andre_REVpri**	D-DNA	André *assay* reverse primer (301 or 70 base-pair amplicons)	N/A	GTGGCCACCGACACCATCT
**TARGETrpoB_WT**	D-DNA	Wild-type rifampicin-susceptible target	N/A	GATGTGCCCGATCGAAACCCCTGAGGGGCCCAACATCGGTCT<br>GATCGGCTCGCTGTCGGTGTACGCGCGGGTCAACCCGTTCGG<br>GTTCATCGAAACGCCGTACCGCAAGGTGGTCGACGGCGTGGT<br>TAGCGACGAGATCGTGTACCTGACCGCCGACGAGGAGGACCG<br>CCACGTGGTGGCACAGGCCAATTCGCCGATCGATGCGGACGG<br>TCGCTTCGTCGAGCCGCGCGTGCTGGTCCGCCGCAAGGCGGG<br>CGAGGTGGAGTACGTGCCCTCGTCTGAGGTGGACTACATGGA<br>CGTCTCGCCCCGCCAGATGGTGTCGGTGGCCAC
**TARGETrpoB_I491F**	D-DNA	I491F mutant target	A1471T	GATGTGCCCGATCGAAACCCCTGAGGGGCCCAACATCGGTCT<br>GTTCGGCTCGCTGTCGGTGTACGCGCGGGTCAACCCGTTCGG<br>GTTCATCGAAACGCCGTACCGCAAGGTGGTCGACGGCGTGGT<br>TAGCGACGAGATCGTGTACCTGACCGCCGACGAGGAGGACCG<br>CCACGTGGTGGCACAGGCCAATTCGCCGATCGATGCGGACGG<br>TCGCTTCGTCGAGCCGCGCGTGCTGGTCCGCCGCAAGGCGGG<br>CGAGGTGGAGTACGTGCCCTCGTCTGAGGTGGACTACATGGA<br>CGTCTCGCCCCGCCAGATGGTGTCGGTGGCCAC
**TARGETrpoB_I491N**	D-DNA	I491N mutant target	T1472A	GATGTGCCCGATCGAAACCCCTGAGGGGCCCAACATCGGTCT<br>GAACGGCTCGCTGTCGGTGTACGCGCGGGTCAACCCGTTCGG<br>GTTCATCGAAACGCCGTACCGCAAGGTGGTCGACGGCGTGGT<br>TAGCGACGAGATCGTGTACCTGACCGCCGACGAGGAGGACCG<br>CCACGTGGTGGCACAGGCCAATTCGCCGATCGATGCGGACGG<br>TCGCTTCGTCGAGCCGCGCGTGCTGGTCCGCCGCAAGGCGGG<br>CGAGGTGGAGTACGTGCCCTCGTCTGAGGTGGACTACATGGA<br>CGTCTCGCCCCGCCAGATGGTGTCGGTGGCCAC
**TARGETrpoB_I491M**	D-DNA	I491M mutant target	C1473A	GATGTGCCCGATCGAAACCCCTGAGGGGCCCAACATCGGTCT<br>GATAGGCTCGCTGTCGGTGTACGCGCGGGTCAACCCGTTCGG<br>GTTCATCGAAACGCCGTACCGCAAGGTGGTCGACGGCGTGGT<br>TAGCGACGAGATCGTGTACCTGACCGCCGACGAGGAGGACCG<br>CCACGTGGTGGCACAGGCCAATTCGCCGATCGATGCGGACGG<br>TCGCTTCGTCGAGCCGCGCGTGCTGGTCCGCCGCAAGGCGGG<br>CGAGGTGGAGTACGTGCCCTCGTCTGAGGTGGACTACATGGA<br>CGTCTCGCCCCGCCAGATGGTGTCGGTGGCCAC

Selecting Susceptible Melt Probe Sequence

A key component of the SMASH assay was the drug-susceptible melt probe, designed as the reverse complement of the drug-susceptible *rpoB* sequence. The probe sequence was selected to maximize the melt temperature (T_m) difference between its duplex with a wild-type *rpoB* strand and its duplex with an I491F variant strand (**Figure S1**).

To optimize this melt difference, we systematically varied the probe's 5' end while keeping its 3' end constant (**Table S2**). Four probe sets (A–D) were designed, progressively increasing the offset between the I491F mutation (nucleotide 1471) and the probe's 5' end. This offset grew one at a time from two bases in Set A (27 bases long) to five bases in Set D (30 bases long, **Table S2**). Prior studies suggest that increasing the mismatch offset destabilizes the duplex more significantly, leading to greater melt shifts[1,2].

For preliminary characterization, we used unblocked melt probe mimics. Melt analysis was performed by comparing the T_m differences between the susceptible melt probe mimic (reverse complement to wild-type *rpoB*) hybridized with either wild-type *rpoB* or I491F asymmetric PCR excess product strand mimics (*rpoB* as is). Each probe set (A–D) was tested with corresponding product strands of increasing length and offset (**Table S2**).

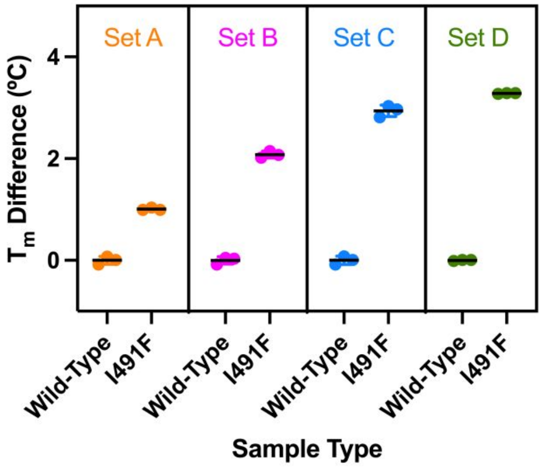

Figure S1. Comparison of melt differences between susceptible melt probe mimics duplexed to either wild-type *rpoB* or I491F variant using the QuantStudio™ 5. Four probe sets (A–D) were tested with corresponding product strands of increasing length and offset (**Table S2**) of *rpoB* nucleotide 1471. T_m differences were calculated between each sample's duplex T_m and the average T_m of duplexed melt probe to wild-type for that particular set. Consistent with duplex destabilization principles, increasing the mismatch offset (across Set A to Set D) led to larger melt shifts between the probe–I491F duplex and the probe–wild-type duplex.

Consistent with duplex destabilization principles[1,2], increasing the mismatch offset led to larger melt shifts between the probe–I491F duplex and the probe–wild-type duplex (**Figure S1**). T_m differences were calculated between each sample's duplex T_m and the average T_m of duplexed melt probe to wild-type for that particular set. All four sets had average T_m differences of 0 ºC for susceptible melt probe bound to wild-type *rpoB*. Average T_m difference for susceptible melt probe bound to I491F increased progressively across sets: 1.01 ºC for Set A (27-bases long, 2-base offset), 2.08 ºC for Set B (28-bases long, 3-base offset), 2.94 ºC for Set C (29-bases long, 4-base offset), and 3.28 ºC for Set D (30-bases long, 5-base offset). Since Set D exhibited the largest T_m difference for I491F discrimination, its probe sequence (SMASH_SetD_mimicSusMeltProbe) was selected for final assay development. This optimized probe (C3-blocked susceptible melt probe, SMASH_SusMeltProbeC3; **Table S1**) facilitated clear discrimination between I491F samples and susceptible melt profiles (**Figure 1A** and **Figure 2A**). Similarly, distinct melt differences were observed for I491N and I491M mismatches (**Figure 1A** and **Figure 2A**).

Methods: The melt probe characterization studies were performed using the QuantStudio™ 5 real-time PCR instrument (Thermo Fisher Scientific #A28137). Reactions had a 20 µL final volume containing 1X of SensiFAST™ Probe No-ROX Kit (Bioline #BIO-86005), 1X LCGreen® Plus (BioFire® Defense, LLC #BCHM-ASY-005), 7.525×10^{12} copies of susceptible melt probe (wild-type reverse complement), and 7.525×10^{12} copies of single-stranded target (either *rpoB* wild-type or I491F mutant). Set A reactions included melt probe *SMASH_SetA_mimicSusMeltProbe* bound to wild-type target *SMASH_SetA_mimicAsymPCRprod_WT* or I491F target *SMASH_SetA_mimicAsymPCRprod_I491F*. Set A oligonucleotides were 27-bases in length and had a 2-base offset of *rpoB* nucleotide 1471. Set B reactions included melt probe *SMASH_SetB_mimicSusMeltProbe* bound to wild-type target *SMASH_SetB_mimicAsymPCRprod_WT* or I491F target *SMASH_SetB_mimicAsymPCRprod_I491F*. Set B oligonucleotides were 28-bases in length and had a 3-base offset of *rpoB* nucleotide 1471. Set C reactions included melt probe *SMASH_SetC_mimicSusMeltProbe* bound to wild-type target *SMASH_SetC_mimicAsymPCRprod_WT* or I491F target *SMASH_SetC_mimicAsymPCRprod_I491F*. Set C oligonucleotides were 29-bases in length and had a 4-base offset of *rpoB* nucleotide 1471. Set D reactions included melt probe *SMASH_SetD_mimicSusMeltProbe* bound to wild-type target *SMASH_SetD_mimicAsymPCRprod_WT* or I491F target *SMASH_SetD_mimicAsymPCRprod_I491F*. Set D oligonucleotides were 30-bases in length and had a 5-base offset of *rpoB* nucleotide 1471.

The high resolution melt was initiated with a 95 °C hold for 2 min followed by annealing 95 ºC to 50 ºC at 0.1 ºC/sec followed by melting 60 ºC to 95 ºC at 0.025 ºC/sec (continuous acquisition mode). Double-stranded DNA fluorescence was monitored during the melt reaction using LCGreen® Plus on the green optical channel (excitation 470±15 / emission 520±15).

Table S2. Oligonucleotide sequences designed for preliminary susceptible melt probe sequence design in the SMASH assay. All sequences are synthesized with D-DNA. <u>Underlining</u> indicates *rpoB* nucleotide 1471 (the SNV location for I491F). Single-base changes for *rpoB* I491F mutants are indicated in red.

ID	Description	Offset of Nucleotide 1471 (from 3' End of Asymmetric PCR Product Mimics and from 5' End of Melt Probe Mimics)	Sequence (5' → 3')
**SMASH_SetA_mimicSusMeltProbe**	Set A susceptible melt probe mimic as reverse complement of wild-type *rpoB* (27 bases in length)	2	GA<u>T</u>CAGACCGATGTTGGGCCCCTCAGG
**SMASH_SetA_mimicAsymPCRprod_WT**	Set A asymmetric PCR product mimic as wild-type *rpoB* (27 bases in length)	2	CCTGAGGGGCCCAACATCGGTCTG<u>A</u>TC
**SMASH_SetA_mimicAsymPCRprod_I491F**	Set A asymmetric PCR product mimic as *rpoB* I491F variant (27 bases in length)	2	CCTGAGGGGCCCAACATCGGTCTG<u>T</u>TC
**SMASH_SetB_mimicSusMeltProbe**	Set B susceptible melt probe mimic as reverse complement of wild-type *rpoB* (28 bases in length)	3	CGA<u>T</u>CAGACCGATGTTGGGCCCCTCAGG
**SMASH_SetB_mimicAsymPCRprod_WT**	Set B asymmetric PCR product mimic as wild-type *rpoB* (28 bases in length)	3	CCTGAGGGGCCCAACATCGGTCTG<u>A</u>TCG
**SMASH_SetB_mimicAsymPCRprod_I491F**	Set B asymmetric PCR product mimic as *rpoB* I491F variant (28 bases in length)	3	CCTGAGGGGCCCAACATCGGTCTG<u>T</u>TCG
**SMASH_SetC_mimicSusMeltProbe**	Set C susceptible melt probe mimic as reverse complement of wild-type *rpoB* (29 bases in length)	4	CCGA<u>T</u>CAGACCGATGTTGGGCCCCTCAGG
**SMASH_SetC_mimicAsymPCRprod_WT**	Set C asymmetric PCR product mimic as wild-type *rpoB* (29 bases in length)	4	CCTGAGGGGCCCAACATCGGTCTG<u>A</u>TCGG
**SMASH_SetC_mimicAsymPCRprod_I491F**	Set C asymmetric PCR product mimic as *rpoB* I491F variant (29 bases in length)	4	CCTGAGGGGCCCAACATCGGTCTG<u>T</u>TCGG
**SMASH_SetD_mimicSusMeltProbe**	Set D susceptible melt probe mimic as reverse complement of wild-type *rpoB* (30 bases in length)	5	GCCGA<u>T</u>CAGACCGATGTTGGGCCCCTCAGG
**SMASH_SetD_mimicAsymPCRprod_WT**	Set D asymmetric PCR product mimic as wild-type *rpoB* (30 bases in length)	5	CCTGAGGGGCCCAACATCGGTCTG<u>A</u>TCGGC
**SMASH_SetD_mimicAsymPCRprod_I491F**	Set D asymmetric PCR product mimic as *rpoB* I491F variant (30 bases in length)	5	CCTGAGGGGCCCAACATCGGTCTG<u>T</u>TCGGC

Tuning Susceptible Melt Probe Concentration

Classifying susceptibility with the SMASH assay relied on a within-sample melt difference between susceptible double-stranded L-DNA and duplexed susceptible melt probe to wild-type asymmetric PCR product. The L-DNA and probe-product duplex had nearly identical melt characteristics when the sequences matched (wild-type *rpoB*, top left panels in **Figure 1B** and **Figure 2B**) but differed if there was a sequence mismatch (*rpoB* I491 variants, top right and lower two panels in **Figure 1B** and **Figure 2B**). Although not critical, melt matching made susceptibility classification visually apparent from the melt curve. To ensure matching melt characteristics between susceptible L-DNA and duplexed susceptible melt probe to wild-type asymmetric PCR product, preliminary experiments were performed varying melt probe strand concentration. Previous reports have established that total DNA concentration and strand ratio affect the melt temperature[3–6]. Prior L-DNA-based work has also employed strand concentration tuning strategies to achieve melt matching for a susceptible L-DNA melt comparator[7]. In the present study, the susceptible melt probe concentration per reaction was reduced such that the T_m of the duplexed susceptible melt probe to wild-type asymmetric PCR product matched the average T_m of the double-stranded L-DNA.

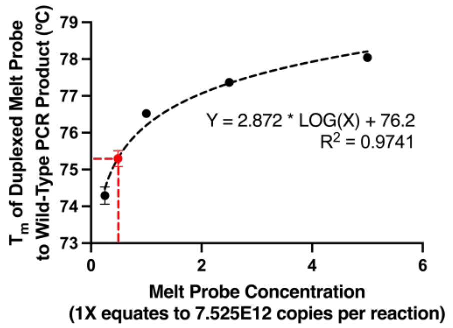

Figure S2. There is a positive logarithmic relationship (dashed black line) between susceptible melt probe concentration per reaction and T_m of duplexed melt probe to wild-type asymmetric PCR product (measured via intercalation signal, n=1 trial in triplicate per concentration). This relationship suggested that a melt probe concentration of 3.665 × 10^{12} copies per reaction (0.487X) would match (dashed red lines) the T_m of duplexed probe and wild-type product to the susceptible L-DNA average T_m of 75.30 ºC (n=1 trial in triplicate across wild-type, variants I491F/N/M, and NTC sample types).

As melt probe concentration per reaction was lowered, probe–wild-type product duplex T_m decreased (**Figure S2**). This phenomenon was used to achieve an empirical melt match between drug-susceptible L-DNA and the duplexed probe to wild-type asymmetric PCR product (top left panels in **Figure 1B** and **Figure 2B**). A positive logarithmic relationship between the number of susceptible melt probe copies per reaction and probe–wild-type duplex T_m indicated that decreasing probe concentration down to 0.487X, or 3.665 × 10^{12} copies per reaction, would produce the 75.30 ºC T_m aligned with the average T_m of susceptible L-DNA (**Figure S2**). The optimal L-DNA strand ratio was rounded up from 0.487X (3.665 × 10^{12} melt probe copies per reaction) to 0.5X (3.7625 × 10^{12} melt probe copies per reaction) for ease of sample preparation in the SMASH assay. This method produced near-zero melt differences between drug-susceptible L-DNA and duplexed melt probe bound to wild-type asymmetric PCR product. Specifically, the average melt difference was -0.23±0.21 ºC using the QuantStudio™ 5 (mean±SD, **Figure 1A**) and -0.08±0.21 ºC using the Rotor-Gene® Q (mean±SD, **Figure 2A**). It is important to note that although this study sought to match T_ms of the L-DNA and the probe–wild-type duplex, it is not critical to do so. Even without tuning the melt probe concentration to make the melt difference zero, for a fixed concentration of melt probe in every sample, the T_m difference will still be a constant in the system and samples can be classified as drug-susceptible when sample T_m difference equals that constant.

Methods: In experiments varying melt probe concentrations, reaction component deviations from standard SMASH assay reactions included 1.881 × 10^{12} (0.25X), 3.763 × 10^{12} (0.5X), 7.525 × 10^{12} (1X), 1.881 × 10^{13} (2.5X), and 3.763 × 10^{13} (5X) copies of susceptible melt probe per reaction. The concentrations were selected using 1X as 7.525 × 10^{12} copies per reaction because this copy number matched the theoretical amount of PCR copies formed by the excess primer at 250 nM per reaction. Tuning experiments were performed in the QuantStudio™ 5 and the only sample type tested was wild-type *rpoB* (n=1 trial in triplicate per melt probe concentration). Logarithmic interpolation of four different melt probe concentrations were used to determine the relationship between copies of melt probe per reaction and melt measurement of duplexed probe to wild-type asymmetric PCR product. The susceptible melt probe copy number with a melt measurement matching that of average susceptible L-DNA was selected. The drug-susceptible L-DNA average T_m was collected across n=1 trial in triplicate across NTC, wild-type, and variant I491F/N/M sample types.

PCR Results

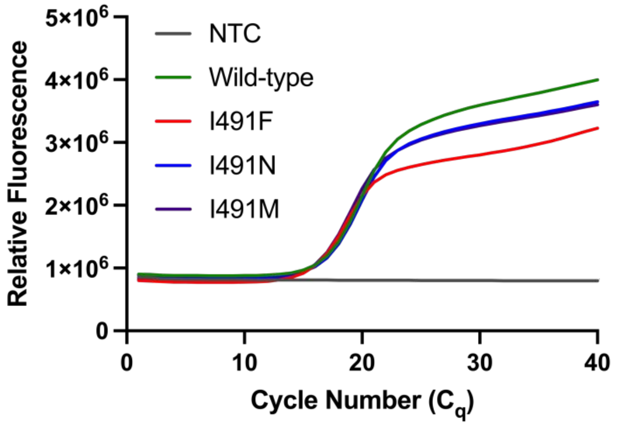

Figure S3. Representative PCR amplification curves of samples tested using the SMASH assay on the QuantStudio™ 5 across wild-type *rpoB*, three *rpoB* variants, and NTC sample types.

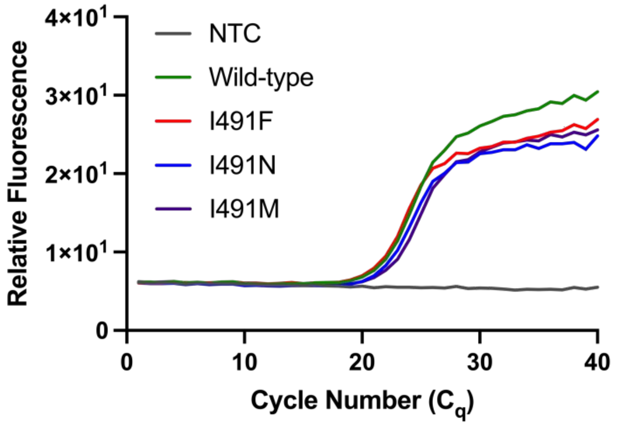

Figure S4. Representative PCR amplification curves of samples tested using the SMASH assay on the Rotor-Gene® Q across wild-type *rpoB*, three *rpoB* variants, and NTC sample types.

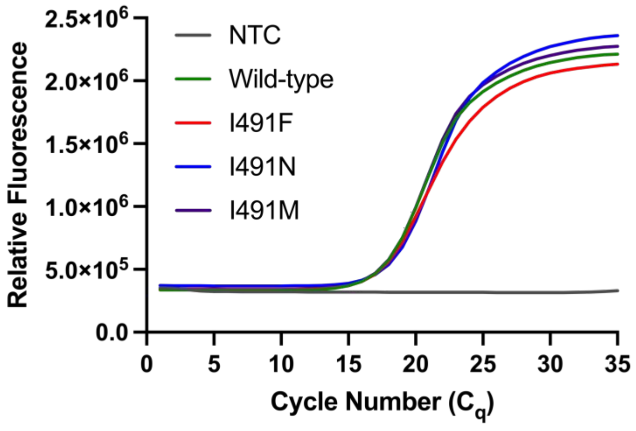

Figure S5. Representative PCR amplification curves of samples tested using the André I491F assay on the QuantStudio™ 5 across wild-type *rpoB*, three *rpoB* variants, and NTC sample types.

Comparing SMASH Assay Melt Curves of NTC Samples Using iFRET Versus Intercalation Alone

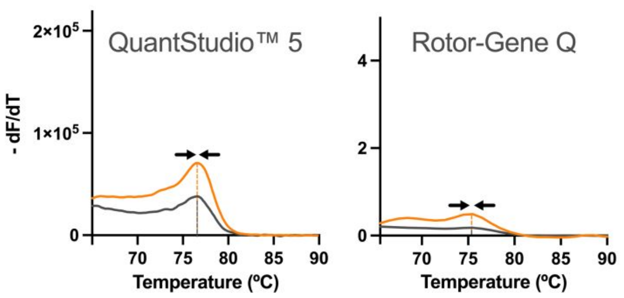

Figure S6. Post-PCR melt curves of representative no template control (NTC) samples produced by the SMASH assay using the QuantStudio™ 5 (left) and Rotor-Gene® Q (right) real-time PCR instruments. Samples contained double-stranded susceptible L-DNA and unbound susceptible melt probe (because no target was present in NTC samples). For each instrument, L-DNA had nearly identical T_m's measured using iFRET signal on the orange optical channel (orange) and using intercalation signal on the green optical channel (gray) within a single-sample. Both instruments demonstrated higher magnitude of fluorescent outputs when the melt curves were produced using iFRET (orange) versus intercalation alone (gray). Dashed vertical lines indicate the T_m's within each sample for L-DNA using iFRET (orange) and intercalation alone (gray) on the horizontal axis.

ROC Analysis Using Within-Sample T_m Differences on SMASH Sample Sets

Table S3. Receiver Operating Characteristic (ROC) susceptibility cutoff values with their associated sensitivity and specificity values from ROC analysis of the SMASH assay across both the QuantStudio™ 5 and Rotor-Gene® Q sample sets using within-sample T_m difference metrics. Since true positives were known, the SMASH assay was assessed for its sensitivity and specificity using ROC analysis (Wilson/Brown method). Across both instruments' sample sets, a T_m difference cutoff point was selected by prioritizing maximized specificity (to decrease the false positive rate, i.e., decrease the misdiagnosis of variant samples as drug-susceptible), followed by maximized sensitivity, when classifying each test sample as drug-susceptible or not. The row highlighted in green designates the selected ROC cutoff point and its associated sensitivity and specificity. Samples were classified as rifampicin-susceptible using a within-sample T_m difference less than 0.83 ºC for the QuantStudio™ 5 and Rotor-Gene® Q sample sets (n=6 trials in triplicate per sample type per instrument).

ROC Cutoff Values (Not susceptible if T_m difference > x)	Sensitivity (%)	Specificity (%)
> -0.54	100	2.8
> -0.48	100	8.3
> -0.43	100	11
> -0.40	100	14
> -0.36	100	17
> -0.33	100	19
> -0.29	100	22
> -0.26	100	31
> -0.23	100	33
> -0.20	100	39
> -0.19	100	50
> -0.16	100	56
> -0.12	100	61
> -0.050	100	67
> 0.10	100	92
> 0.25	100	94
> 0.38	100	97
> 0.83	100	100
> 1.4	99	100
> 1.6	94	100
> 1.8	90	100
> 1.9	85	100
> 2.1	81	100
> 2.2	78	100
> 2.2	69	100
> 2.3	69	100
> 2.3	67	100
> 2.4	66	100
> 2.4	65	100
> 2.4	64	100
> 2.4	63	100
> 2.5	61	100
> 2.5	60	100
> 2.5	55	100
> 2.5	54	100
> 2.5	52	100
> 2.5	51	100
> 2.6	50	100
> 2.6	46	100
> 2.6	45	100
> 2.6	44	100
> 2.7	42	100
> 2.7	36	100
> 2.7	34	100
> 2.7	33	100
> 2.7	31	100
> 2.7	31	100
> 2.7	27	100
> 2.7	25	100
> 2.7	23	100
> 2.7	22	100
> 2.8	19	100
> 2.8	17	100
> 2.8	11	100
> 2.9	10	100
> 2.9	8.3	100
> 2.9	7.4	100
> 2.9	6.5	100
> 3.0	5.6	100
> 3.0	2.8	100
> 3.1	1.9	100
> 3.2	0.93	100

Classification and ROC Analysis Using Multi-Sample Comparison on SMASH Sample Sets

The SMASH assay for *rpob* codon 491 included two indicators of rifampicin susceptibility in every sample: a susceptible melt probe and a susceptible L-DNA. L-DNA offers numerous advantages to the assay, such as providing an internal melt comparison for susceptibility in every sample, correcting for within-assay variability as a hybridization melt standard, and enabling single-sample classification without relying on multi-sample comparison or historical data. However, the iFRET strategy used to acquire L-DNA fluorescence during melt analysis requires that the real-time PCR instrument be capable of excitation in one optical channel and emission in another. Common real-time PCR instruments, like the LightCycler 480[8], QuantStudio™ 3[9], QuantStudio™ 5 [9], and Rotor-Gene® Q[10], meet this criterion. In cases where such instrument capabilities are not available or other L-DNA requirements are not met, the SMASH I491 assay developed in this work can be adapted to be L-DNA-independent.

The original QuantStudio™ 5 and Rotor-Gene® Q datasets, which included both melt comparators (melt probe and L-DNA), were re-analyzed using a multi-sample T_m comparison (**Figure S7**) —similar to the André assay's analysis strategy—rather than a within-sample T_m comparison employed in the main report (**Figure 1A** and **Figure 2A**). Here, T_m difference was calculated as the difference between each sample's probe–product duplex T_m and the sample set's global average T_m of the probe bound to wild-type asymmetric PCR product.

Even without incorporating L-DNA melt data into classification analysis, the SMASH assay still successfully classified all *rpoB* I491 variants (I491F, I491N, and I491M) as not susceptible and all wild-type *rpoB* samples as susceptible (**Figure S7**). The assay maintained high performance across two highly calibrated, real-time PCR platforms, the QuantStudio™ 5 (**Figure S7A**) and the Rotor-Gene® Q (**Figure S7B**). Both PCR systems demonstrated 100% sensitivity and 100% specificity when classifying rifampicin susceptibility (n=6 trials in triplicate per sample type per instrument, **Figure S7**). Successful sample classification is illustrated by 18/18 wild-type *rpoB* samples below the susceptible cutoff and 54/54 variant I491 samples above the susceptible cutoff, in both the QuantStudio™ 5 sample set (**Figure S7A**) and the Rotor-Gene® Q sample set (**Figure S7B**).

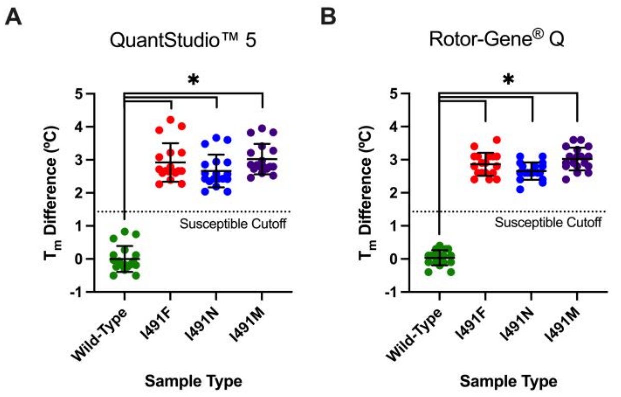

Figure S7. Performance of the SMASH assay without incorporating the L-DNA melt data and instead using multi-sample T_m difference comparison for susceptibility classification. (**A**) The QuantStudio™ 5 sample set assessed for T_m differences between each sample's probe-product duplex T_m and the sample set's global average T_m of probe bound to wild-type product across wild-type (green) and variants I491F (red), I491N (blue), and I491M (purple). Samples were classified as drug-susceptible when sample T_m difference was less than the drug-susceptible cutoff of 1.43 ºC (indicated by dashed black line and determined by ROC cutoff analysis in **Table S4**). Each technical replicate is represented by a single dot. Average T_m difference ± standard deviation per sample type is indicated across n=6 trials in triplicate per sample type. Statistical significance is indicated by solid black lines with asterisks (p<0.0001). (**B**) The Rotor-Gene® Q sample set assessed for T_m differences between each sample's probe–product duplex T_m and the sample set's global average T_m of probe bound to wild-type product across wild-type (green) and variants I491F (red), I491N (blue), and I491M (purple). Samples were classified as drug-susceptible when sample T_m difference was less than the drug-susceptible cutoff of 1.43 ºC (indicated by dashed black line and determined by ROC cutoff analysis in **Table S4**). Each technical replicate is represented by a single dot. Average T_m difference ± standard deviation per sample type is indicated across n=6 trials in triplicate per sample type. Statistical significance is indicated by solid black lines with an asterisk (p<0.0001).

Using ROC analysis (**Table S4**), QuantStudio™ 5 and Rotor-Gene® Q samples were classified as rifampicin-susceptible using a T_m difference less than 1.43 ºC. The QuantStudio™ 5 sample set's average T_m differences for wild-type, I491F, I491N, and I491M were 0.00±0.39, 2.92±0.58, 2.66±0.50, and 3.02±0.46, respectively (mean±SD). Using melt difference comparison amongst the QuantStudio™ 5 sample set, there was a significant difference between wild-type and I491F (p<0.0001) but no significant difference between wild-type and I491N or wild-type and I491M (p>0.05, one-way ANOVA, Tukey's post hoc test for multiple comparisons, 9 replicates per sample type, **Figure S7A**). The Rotor-Gene® Q sample set's average T_m differences for wild-type, I491F, I491N, and I491M were 0.03±0.23, 2.86±0.35, 2.66±0.26, and 3.02±0.34, respectively (mean±SD). Using melt difference comparison, there was a significant difference between wild-type and I491F (p<0.0001) but no significant difference between wild-type and I491N or wild-type and I491M (p>0.05, one-way ANOVA, Tukey's post hoc test for multiple comparisons, 9 replicates per sample type, **Figure S7B**).

Methods: The analyses of multi-sample melt difference and statistical significance were performed independently for the QuantStudio™ 5 and Rotor-Gene® Q using the primary report's original data sets collected for the SMASH assay. A multi-sample melt difference was calculated between the T_m of each sample's probe–product duplex and the sample set's global average T_m of the probe bound to wild-type product. Significance was evaluated using multi-sample melt difference comparison (one-way ANOVA, Tukey's post hoc test for multiple comparisons, significance level of α=0.95) across I491F, I491N, and I491F as compared to wild-type (n=6 trials in triplicate per sample type per instrument). Since true positives were known, the SMASH assay was assessed for its sensitivity and specificity using Receiver Operating Characteristic (ROC) analysis (Wilson/Brown method) across both the QuantStudio™ 5 and Rotor-Gene® Q sample sets (**Table S4**). Across both instruments' sample sets, a universal T_m difference cutoff point was selected by prioritizing maximized specificity (to decrease the false positive rate, i.e., decrease the misdiagnosis of variant samples as drug-susceptible), followed by maximized sensitivity, when classifying each test sample as drug-susceptible or not. Samples were classified as rifampicin-susceptible using a multi-sample T_m difference less than 1.43 ºC for the QuantStudio™ 5 and Rotor-Gene® Q sample sets (n=6 trials in triplicate per sample type per instrument). All statistics were performed in GraphPad Prism version 10.0.

Table S4. Receiver Operating Characteristic (ROC) susceptibility cutoff values with their associated sensitivity and specificity values from ROC analysis of the SMASH assay across both the QuantStudio™ 5 and Rotor-Gene® Q sample sets using multi-sample T_m difference metrics. Since true positives were known, the SMASH assay was assessed for its sensitivity and specificity using ROC analysis (Wilson/Brown method). Across both instruments' sample sets, a T_m difference cutoff point was selected that maximized specificity (to decrease the false positive rate, i.e., decrease the misdiagnosis of variant samples as drug-susceptible), followed by maximized sensitivity, when classifying each test sample as drug-susceptible or not. The row highlighted in green designates the selected ROC cutoff point and its associated sensitivity and specificity. Samples were classified as rifampicin-susceptible using a multi-sample T_m difference less than 1.43 ºC for the QuantStudio™ 5 and Rotor-Gene® Q sample sets (n=6 trials in triplicate per sample type per instrument).

ROC Cutoff Values (Not susceptible if T_m difference > x)	Sensitivity (%)	Specificity (%)
> -0.451	100	5.56
> -0.368	100	11.1
> -0.286	100	13.9
> -0.218	100	19.4
> -0.195	100	22.2
> -0.181	100	27.8
> -0.136	100	33.3
> -0.0840	100	44.4
> -0.0500	100	50.0
> 0.0340	100	52.8
> 0.109	100	72.2
> 0.196	100	77.8
> 0.287	100	80.6
> 0.350	100	88.9
> 0.508	100	91.7
> 0.681	100	94.4
> 0.786	100	97.2
> 1.43	100	100
> 2.07	98.1	100
> 2.15	97.2	100
> 2.23	96.3	100
> 2.27	95.4	100
> 2.29	94.4	100
> 2.33	93.5	100
> 2.37	92.6	100
> 2.40	91.7	100
> 2.41	86.1	100
> 2.42	85.2	100
> 2.44	81.5	100
> 2.49	80.6	100
> 2.54	79.6	100
> 2.58	78.7	100
> 2.59	76.9	100
> 2.60	75.9	100
> 2.62	62.0	100
> 2.65	61.1	100
> 2.66	59.3	100
> 2.69	57.4	100
> 2.72	56.5	100
> 2.76	54.6	100
> 2.79	53.7	100
> 2.79	51.9	100
> 2.80	50.0	100
> 2.82	42.6	100
> 2.85	41.7	100
> 2.86	40.7	100
> 2.88	39.8	100
> 2.91	33.3	100
> 2.93	32.4	100
> 2.96	31.5	100
> 3.03	30.6	100
> 3.09	29.6	100
> 3.16	19.4	100
> 3.21	18.5	100
> 3.25	16.7	100
> 3.29	15.7	100
> 3.35	14.8	100
> 3.41	12.0	100
> 3.45	11.1	100
> 3.54	10.2	100
> 3.60	7.41	100
> 3.64	6.48	100
> 3.75	5.56	100
> 3.83	4.63	100
> 3.86	3.70	100
> 3.92	2.78	100
> 3.99	1.85	100
> 4.12	0.926	100

References for Supporting Information

(1) Cisse, I. I.; Kim, H.; Ha, T. A Rule of Seven in Watson-Crick Base-Pairing of Mismatched Sequences. *Nat Struct Mol Biol* **2012**, *19* (6), 623–627. https://doi.org/10.1038/nsmb.2294.

(2) Little, S. Amplification-Refractory Mutation System (ARMS) Analysis of Point Mutations. *Curr Protoc Hum Genet* **1995**, *Chapter 9* (1), Unit 9.8. https://doi.org/10.1002/0471142905.hg0908s07.

(3) Schreiber-Gosche, S.; Edwards, R. A. Thermodynamics of Oligonucleotide Duplex Melting. *J Chem Educ* **2009**, *86* (5), 644–650. https://doi.org/10.1021/ED086P644.

(4) Wu, P.; Nakano, S. I.; Sugimoto, N. Temperature Dependence of Thermodynamic Properties for DNA/DNA and RNA/DNA Duplex Formation. *Eur J Biochem* **2002**, *269* (12), 2821–2830. https://doi.org/10.1046/J.1432-1033.2002.02970.X.

(5) You, Y.; Tataurov, A. V.; Owczarzy, R. Measuring Thermodynamic Details of DNA Hybridization Using Fluorescence. *Biopolymers* **2011**, *95* (7), 472. https://doi.org/10.1002/BIP.21615.

(6) Spurlock, N.; Gabella, W. E.; Nelson, D. J.; Evans, D. T.; Pask, M. E.; Schmitz, J. E.; Haselton, F. R. Implementing L-DNA Analogs as Mirrors of PCR Reactant Hybridization State: Theoretical and Practical Guidelines for PCR Cycle Control. *Anal Methods* **2024**, *16* (18), 2769–2974. https://doi.org/10.1039/D4AY00083H.

(7) Malofsky, N. A.; Nelson, D. J.; Pask, M. E.; Haselton, F. R. L-DNA-Based Melt Analysis Enables Within-Sample Validation of PCR Products. *Anal Chem* **2024**, *96* (29), 11897–11905. https://doi.org/10.1021/acs.analchem.4c01611.

(8) Roche Diagnostics GmbH. *LightCycler ® 480 Instrument Operator's Manual, Software Version 1.5*; 2008. http://www.roche-applied-science.com/ (accessed 2025-01-27).

(9) Thermo Fisher Scientific Inc. QuantStudio 3 and 5 Real-Time PCR Systems Installation, Use, and Maintenance Guide. **2021**.

(10) Qiagen. *Rotor-Gene Q User Manual*; 2023.
